# Cell Wall Anchoring of a Bacterial Chitosanase in *Lactobacillus plantarum* Using a Food-Grade Expression System and Two Versions of an LP × TG Anchor

**DOI:** 10.3390/ijms21113773

**Published:** 2020-05-27

**Authors:** Mai-Lan Pham, Anh-Minh Tran, Geir Mathiesen, Hoang-Minh Nguyen, Thu-Ha Nguyen

**Affiliations:** 1 Food Biotechnology Laboratory, Department of Food Science and Technology, BOKU-University of Natural Resources and Life Sciences, Vienna, Muthgasse 18, 1190 Vienna, Austria; mailanpham.22@gmail.com (M.-L.P.); anh.tran@boku.ac.at (A.-M.T.); 2 Department of Biology, Faculty of Fundamental Sciences, Ho Chi Minh City University of Medicine and Pharmacy, 217 Hong Bang, Ho Chi Minh City, Vietnam; 3 Faculty of Chemistry, Biotechnology and Food Science, Norwegian University of Life Sciences (NMBU), P.O. Box 5003, N-1432 Ås, Norway; geir.mathiesen@nmbu.no; 4 Department of Biotechnology, The University of Danang-University of Science and Technology, 54 Nguyen Luong Bang, Da Nang 550000, Vietnam

**Keywords:** surface display, cell wall anchor, *Lactobacillus plantarum*, whole-cell biocatalyst

## Abstract

Lactic acid bacteria (LAB) have attracted increasing interest recently as cell factories for the production of proteins as well as a carrier of proteins that are of interest for food and therapeutic applications. In this present study, we exploit a lactobacillal food-grade expression system derived from the pSIP expression vectors using the *alr* (alanine racemase) gene as the selection marker for the expression and cell-surface display of a chitosanase in *Lactobacillus plantarum* using two truncated forms of a LP × TG anchor. CsnA, a chitosanase from *Bacillus subtilis* 168 (ATCC23857), was fused to two different truncated forms (short-S and long-L anchors) of an LP × TG anchor derived from Lp_1229, a key-protein for mannose-specific adhesion in *L. plantarum* WCFS1. The expression and cell-surface display efficiency driven by the food-grade *alr*-based system were compared with those obtained from the *erm-*based pSIP system in terms of enzyme activities and their localisation on *L. plantarum* cells. The localization of the protein on the bacterial cell surface was confirmed by flow cytometry and immunofluorescence microscopy. The highest enzymatic activity of CsnA-displaying cells was obtained from the strain carrying the *alr*-based expression plasmid with short cell wall anchor S. However, the attachment of chitosanase on *L. plantarum* cells via the long anchor L was shown to be more stable compared with the short anchor after several repeated reaction cycles. CsnA displayed on *L. plantarum* cells is catalytically active and can convert chitosan into chito-oligosaccharides, of which chitobiose and chitotriose are the main products.

## 1. Introduction

Chitin, which can be found in the outer exoskeleton of arthropods, such as crabs, lobster, shrimp, and in the fungal cell wall, is the second most abundant biopolymer after lignocellulose in nature [1]. An enormous amount of chitin is annually disposed into the sea, which requires a need for bioremediation or recycling of the chitin biomass [2]. Chitosan is a natural nontoxic biopolymer, which can be obtained from the partially de-acetylation of chitin under alkaline conditions and is composed of β-1,4-*N*-acetyl-D-glucosamine (GlcNac) and D-glucosamine (GlcN) [3]. Despite its biological activities, its applications were limited due to its high molecular weight and solubility at acidic pH [4]. However, the depolymerisation products from chitosan, so-called chito-oligosaccharides (CHOS), which possess potential properties for pharmaceutical and medical applications, are of interest due to their nontoxic and high solubility properties [3,4]. CHOS can be produced by enzymatic hydrolysis using chitosanases or chitosan *N*-acetyl-glucosaminohydrolases (EC3.2.1.132) [3,5]. These enzymes catalyse the endohydrolysis β-1,4-glycosidic bond between D-glucosamine of chitosan and have been found in various organisms including Gram-positive bacteria, Gram-negative bacteria, and fungi [5,6,7,8,9]. Among them, the chitosanases from family GH46, especially *Bacillus subtilis* 168, showed the potential for bioconversion of chitosan to produce CHOS [5,10]. The chitosanase from *B. subtilis* 168, which is encoded by *csnA* gene, was demonstrated to be efficiently produced and secreted in *Lactobacillus plantarum* using the inducible promoter-based pSIP expression vectors containing either an antibiotic resistance or the *alr* gene as selection markers [11].

Recently, bacterial cell-surface display has become an attractive strategy for the development of whole-cell biocatalysts. By fusing with an anchoring motif, the protein of interest can be simultaneously synthesized and subsequently displayed on the bacterial cells. Obtained cells harbouring fusion target protein/enzyme after fermentation can be directly used as biocatalysts for the reaction processes. This concept offers known advantages such as the immobilisation of enzymes with a significant reduction of production cost and utilisation of the bacterial biomass as the immobilisation matrix instead of traditional carrier materials. With a long history of use in food industry and possession of GRAS (generally regarded as safe) status, *L. plantarum* has been exploited as host for cell-surface display of heterologous proteins [12,13,14,15,16,17,18,19,20,21]. In our previous study, we reported cell-surface display of a chitosanase via a lipoprotein anchor (Lp_1261) and an LP × TG motif-containing a cell wall anchor from a cell surface adherence protein (Lp_2578) of *L. plantarum* WCFS1 [22], using the original *erm*-based pSIP expression system [23,24]. It was shown that surface display of the chitosanase in lactobacilli using the Lp_2578 derived cell wall anchor resulted in higher enzyme activity than *Lactobacillus* strains with Lp_1261 derived lipoprotein anchor [22].

Nevertheless, the use of antibiotic resistance gene as the selection marker in original pSIP vectors [23,25], which requires the supplement of erythromycin in cultivation media, might lead to the limitation of this system in some applications, e.g., production of food ingredients and additives. Therefore, a food-grade selection marker based on complementation would be a more useful alternative [26]. We have previously developed a food-grade, complementation-based host/marker expression system derived from the pSIP expression vectors using the *alr* (alanine racemase) gene as the selection marker for intracellular expression of β-galactosidases in *L. plantarum* [27]. In the present study, we exploit this food-grade expression system for the expression and cell-surface display of a chitosanase derived from *B. subtilis* 168 (ATCC23857) in *L. plantarum*. We have constructed two variants of LP × TG anchor derived from Lp_1229 with different lengths of the linker between the anchoring motif and the chitosanase to investigate the effect of anchor length on the enzymatic activity of chitosanase displaying cells. The expression and cell-surface display efficiency driven by the food-grade system were compared with those obtained from the *erm-*based pSIP system in term of enzyme activities and their localisation on *L. plantarum* cells.

## 2. Results

### 2.1. Expression of Chitosanase (CsnA) in L. plantarum

**Figure 1:**
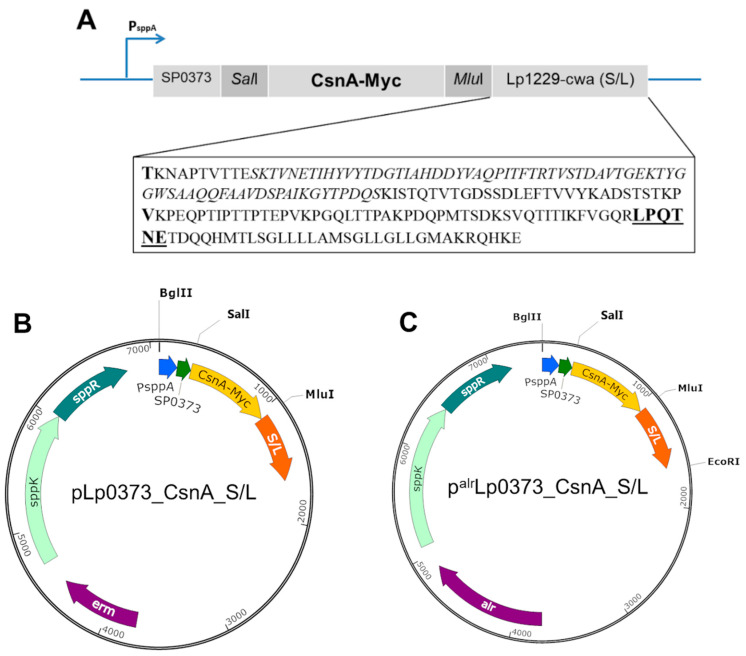
The expression cassette (**A**), *erm*-based and *alr*-based expression vectors (**B**,**C**) of C-terminal cell-wall anchor of chitosanase (CsnA) in *L. plantarum*. (**A**) Myc tag sequence was fused to the C-terminus of CsnA for protein detection. All parts of the expression cassette can be easily exchangeable using the indicated restriction enzymes (*Bgl*II, *Sal*I, *Mul*I, *EcoR*I). The signal peptide (SP) derived from Lp_0373 was fused to CsnA via *Sal*I site. The short anchor S (85 residues) consists of a linker of 47 residues between the LP × TG motif, which is the consensus sequence LPQTNE in *L. plantarum* [30,31] (bold, underlined), and the C-terminus of CsnA-Myc with a valine as the fusing point (V in bold). The long anchor L (194 residues) contains a linker of 156 residues upstream of the LP × TG motif including one Mub B2-like domain of 69 residues (mucus binding domain; in italics) fused to CsnA-Myc with a threonine residue (T in bold). (**B**) Schematic overview of the plasmid encoding CsnA fused to a LP × TG cell wall anchor using different lengths of Lp_1229 derived anchor and Lp_0373 derived SP for secretion. The plasmid contains an erythromycin antibiotic resistance gene (*erm*) as selection marker. (**C**) Schematic overview of the plasmid encoding CsnA fused to a LP × TG cell wall anchor using different lengths of Lp_1229 derived anchor and Lp_0373 derived SP for secretion. The plasmid contains alanine racemase gene (*alr*) as selection marker. See text for more details.

Four new expression vectors were constructed to display a chitosanase from *Bacillus subtilis* 168 on the surface of *L. plantarum* (Figure 1A–C) using both *erm*-based and *alr*-based expression systems (erythromycin and alanine racemase as selection markers, respectively) in lactobacilli. The enzyme was C-terminally anchored to the cell wall using two different truncated forms, namely S (Short) and L (long) anchors, of an LP × TG anchor derived from the Lp_1229 sequence, which encoding a protein containing predicted mucus binding domains in *L. plantarum* [28,29]. For the immunodetection of the recombinant proteins, a Myc-tag was fused C-terminally to the CsnA sequence. The short anchor S (85 residues) consisting of the LP × TG motif, which is the actual consensus sequence LPQTNE in *L. plantarum* [30,31] (in bold, underlined, Figure 1A), and a linker of 47 residues upstream of the LP × TG motif were fused C-terminally to CsnA-Myc sequence using a valine residue (V in bold, Figure 1A). The longer anchor L (194 residues) contains a linker of 156 residues upstream of the LP × TG motif, which consists of one Mub B2-like domain of 69 residues (mucus binding domain; in italics, Figure 1A) [28,32] that is separated from consensus sequence LPQTNE by 77 residues, and 10 residues upstream of Mub B2-like domain (Figure 1A). The CsnA-Myc sequence was fused to L anchor at threonine residue (T in bold, Figure 1A). For efficient secretion, a signal peptide (SP) derived from Lp_0373, which was previously shown to efficiently secrete heterologous proteins in *L. plantarum* [33,34,35], was fused to CsnA through the *Sal*I linker (Figure 1A).

**Figure 2:**
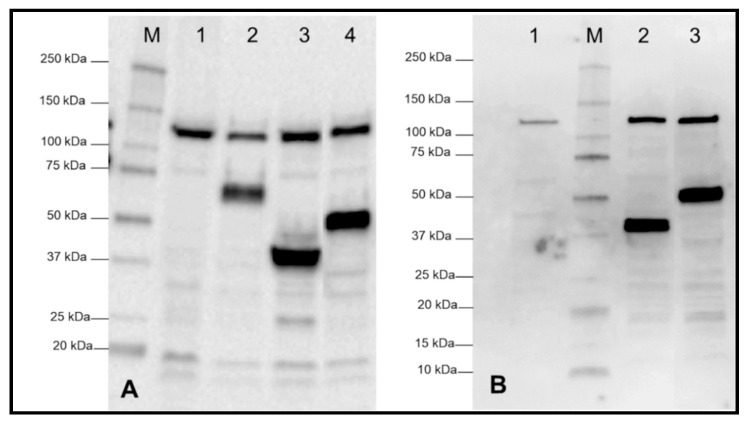
Western blot analysis of cell-free extracts from transformed and induced *L. plantarum* cells harbouring various expression plasmids. (**A**) *erm*-based expression system (1) pEV, empty vector as negative control; (2) pSIP_CsnAcwa2: inducible expression vector for cell wall anchoring of CsnA using cwa2 derived from the Lp_2578 protein as positive control [22]; (3) pLp0373_CsnA_S (expected protein size 39 kDa); (4) pLp0373_CsnA_L (expected protein size 52 kDa). (**B**) *alr*-based expression system (1) *L. plantarum* TLG02 as negative control [27]; (2) p^alr^Lp0373_CsnA_S (expected protein size 39 kDa); (3) p^alr^Lp0373_CsnA_L (expected protein size 52 kDa). M indicates molecular mass markers (Biorad).

All engineered plasmids, pLp0373_CsnA_S/L and p^alr^Lp0373_CsnA_S/L, were then transformed into *L. plantarum* WCFS1 or D-alanine auxotroph strain, *L. plantarum* TLG02 [27], respectively. To analyse the production of the chitosanase, Western blot analysis of the crude, cell-free extracts was performed using anti-Myc antibodies for detection of the enzyme. Figure 2 shows that all four recombinant strains harbouring the plasmids of both expression systems produced the expected proteins. The *L. plantarum* WCFS1 strain harbouring an empty vector (pEV) (lane 1, Figure 2A) and the *L. plantarum* TLG02 strain without plasmid (lane 1, Figure 2B) used as the negative controls did not produce any target protein as expected, while the strain harbouring pSIP_CsnAcwa2 (lane 2, Figure 2A), in which the chitosanase from *B. sutbtilis* 168 is fused to an LP × TG anchor derived from the Lp_2578 (cwa2) [12,22], was used as a positive control. Figure 2A,B show the presence of chitosanases with two cell wall anchors S/L at expected molecular weights of ~39 kDa and ~52 kDa, respectively (Figure 2A, lanes 3–4 for the strains harbouring pLp0373_CsnA_S/L; Figure 2B, lanes 2–3 for strains harbouring p^alr^Lp0373_CsnA_S/L, respectively).

### 2.2. Enzymatic Activity of CsnA-Displaying Cells

**Figure 3:**
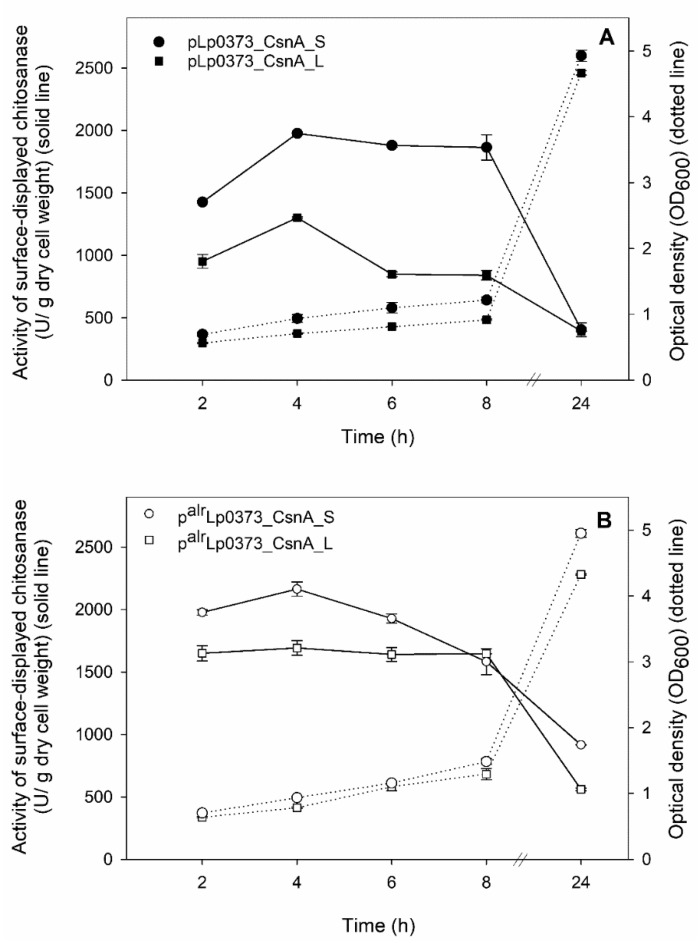
Enzymatic activity of CsnA displaying cells. Time course of cultivations of CsnA-displaying *L. plantarum* recombinant strains harboring the plasmids of *erm*-based expression system (**A**) and *alr*-based expression system (**B**) in MRS medium at 37 °C. Experiments were performed in triplicates, and the standard deviation was always less than 5%.

Chitosanase activities of living recombinant lactobacillal cells were measured using 0.5% (w/v) of chitosan as the substrate to evaluate the functionality of the surface-displayed enzymes. Figure 3A,B shows the time course of the cultivations of CsnA-displaying *L. plantarum* strains harboring *erm*-based and *alr*-based expression plasmids, respectively, and the highest levels of chitosanase-displaying activities (U/g dry cell weight) of recombinant bacteria from both systems were obtained at 4 h after induction (OD_600_ ~ 1.0–1.2). A significant decrease in activities (U/g dry cell weight; U/g DCW) was observed as the cultivation was extended after 8 h of induction. The highest enzymatic activities of CsnA-displaying cells of 1975 U/g DCW and 2160 U/g DCW were obtained from the strains carrying the plasmids with short cell wall anchor S, pLp0373_CsnA_S and p^alr^Lp0373_CsnA_S, respectively (Figure 3A,B). On the other hand, the highest enzymatic activities of CsnA-displaying cells obtained from the strains carrying the plasmids with long cell wall anchor L, pLp0373_CsnA_L and p^alr^Lp0373_CsnA_L, were 1300 U/g DCW and 1690 U/g DCW, respectively (Figure 3A,B). It was also confirmed that the enzymatic activities obtained from CsnA-displaying cells were indeed from surface-anchored chitosanase as no enzymatic activities were detected from *Lactobacillus* cells carrying the empty plasmid pEV (negative control) or from the host strains.

### 2.3. Surface Localization of CsnA in L. plantarum and the Stability of CsnA-Displaying Cells

**Figure 4:**
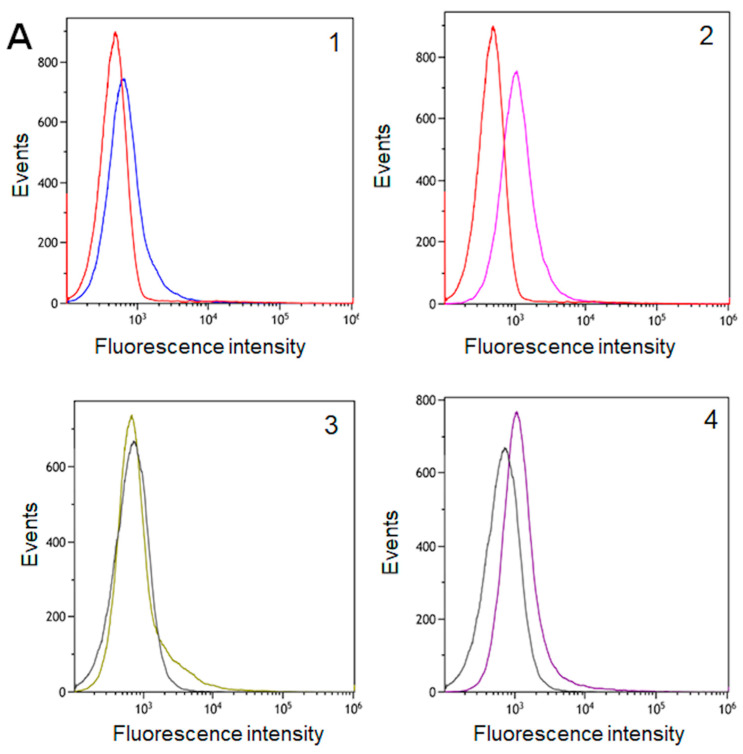

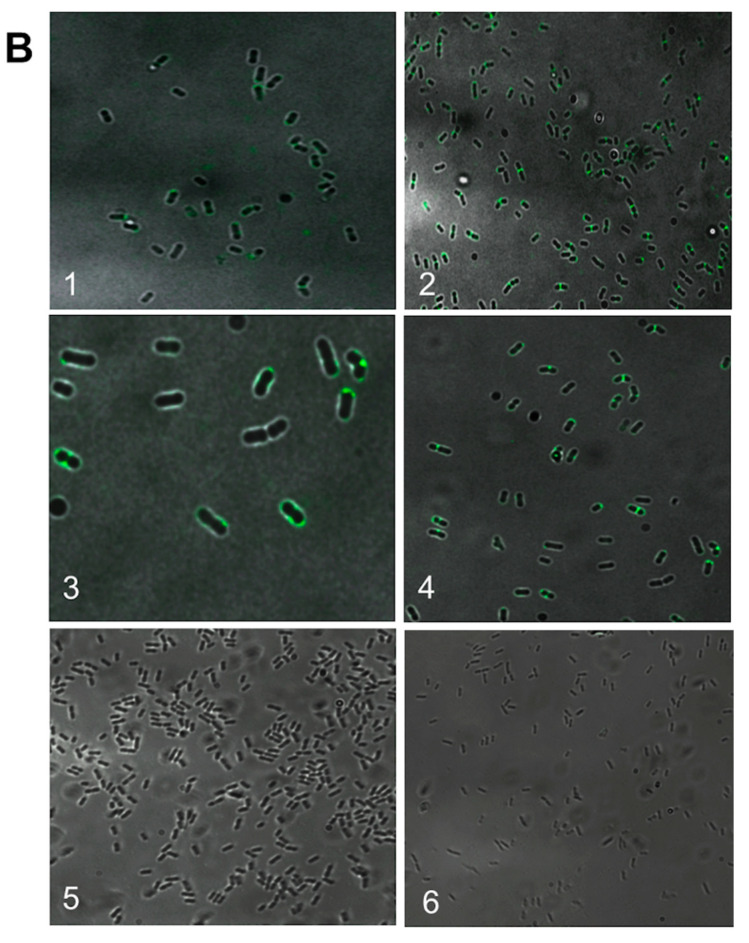
Surface localisation of chitosanase in *L. plantarum* cells analysed by flow cytometry (**A**) and immunofluorescence microscopy (**B**). The *L. plantarum* strains are denoted by different numbers in the flow cytometry histograms (**A**) and in the micrographs (**B**): pLp0373_CsnA_S (**A1**,**B1**); pLp0373_CsnA_L (**A2**,**B2**); p^alr^Lp0373_CsnA_S (**A3**,**B3**); p^alr^Lp0373_CsnA_L (**A4**,**B4**); *L. plantarum* WCFS1 harboring an empty vector pEV (red line in **A1**–**A2**; **B5**) and *L. plantarum* TLG02 (black line in **A3**–**A4**; **B6**) were used as negative controls.

Even though significant enzymatic activities of CsnA-displaying cells were obtained from the strains carrying the plasmids with short cell wall anchor S, only slight shift in the fluorescence signal was detected for the strain harbouring the plasmid of *erm*-based expression system pLp0373_CsnA_S and we could not observe a clear shift in the fluorescence signal for the strain harbouring the plasmid of *alr*-based expression system p^alr^Lp0373_CsnA_S compared to the control strain (Figure 4A). On the other hand, flow cytometry analysis confirmed surface display of the chitosanase for the strains carrying the plasmids with long cell wall anchor L, pLp0373_CsnA_L and p^alr^Lp0373_CsnA_L, as indicated by the clear shifts in the fluorescence signals observed for these strains compared to the negative control strains (Figure 4A). Interestingly, immunofluorescence microscopy clearly confirmed the surface localization of the Myc-tag in all recombinant strains carrying the plasmids with both short and long cell wall anchors. However the number of CsnA-displaying cells from the strains carrying the plasmids with short cell wall anchor S, pLp0373_CsnA_S and p^alr^Lp0373_CsnA_S, were notably lower compared to the strains carrying the plasmids with long cell wall anchor L (Figure 4B), even though the same number of cells in all samples were used at the beginning of the experiments.

**Figure 5:**
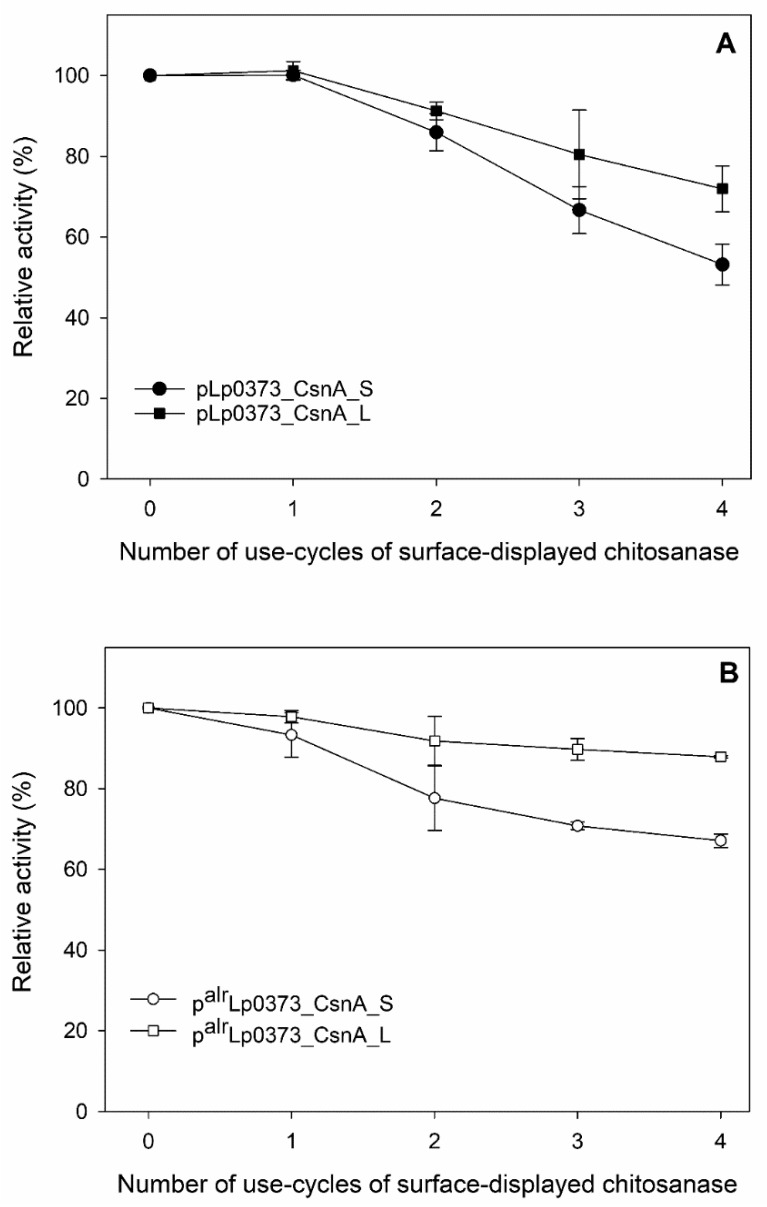
Enzymatic activity of CsnA displaying cells of repeated activity measurements. 0 indicates the first measurement after harvesting, while 1, 2, 3, 4 indicate the number of replications. (**A**) *L. plantarum* cells harbouring the *erm*-based expression plasmids (**B**) *L. plantarum* cells harbouring the *alr*-based expression plasmids. Experiments were performed in triplicates, and the standard deviation was always less than 5%.

These results are supported by the observations of the stability of CsnA-displaying cells. We measured the enzyme activities of surface-displayed chitosanase of *Lactobacillus* cells during four repeated cycles with a washing step between the cycles to remove proteins released from lysed cells. Interestingly, CsnA-displaying cells from the strains carrying the plasmids with short anchor S showed less stable after repeated cycles compared to the strains carrying the plasmids with long anchor L. The enzymatic activities of *Lactobacillus* cells harbouring the plasmids with S anchor, pLp0373_CsnA_S and p^alr^Lp0373_CsnA_S, decreased significantly as indicated by activity losses of ~50% and 35%, respectively, after four assay/washing cycles (Figure 5A,B). The chitosanase displaying cells via L anchor of *erm*- and *alr*-based expression systems retained more than 70% and ~90% after four assay/washing cycles, respectively, of their initial chitosanase activities, indicating that these enzyme-displaying cells can be reused for several rounds of biocatalysis at 37 °C (Figure 5A,B). Hence, the observation that a lower number of cells harbouring pLp0373_CsnA_S and p^alr^Lp0373_CsnA_S exposed the fluorescence signals in immunofluorescence microscopy analysis can be explained by the fact that a high number of cells lost the anchored chitosanase during the preparation of the cells prior to the analysis due to the unstable attachment of the enzyme to the cell wall.

Furthermore, the thermal stability of *L. plantarum* TLG02 cells carrying the plasmid p^alr^Lp_0373_CsnA_L at various temperatures was investigated. It was shown that chitosanase displaying cells are very stable at the storage temperature of −20 °C with a half-life of ~9 months (data not shown). The activity of CsnA-anchored cells was also stable at 37 °C. In fact, the displaying cells lost 50% of initial chitosanase activity only after five weeks. CsnA-displaying cells retained 50% of initial enzyme activity for ~24 h at 50 °C (data not shown), which is of great interest from an application point of view for chitosan conversion.

### 2.4. Chitosan Conversion and Products Analysis by Thin Layer Chromatography (TLC)

**Figure 6:**
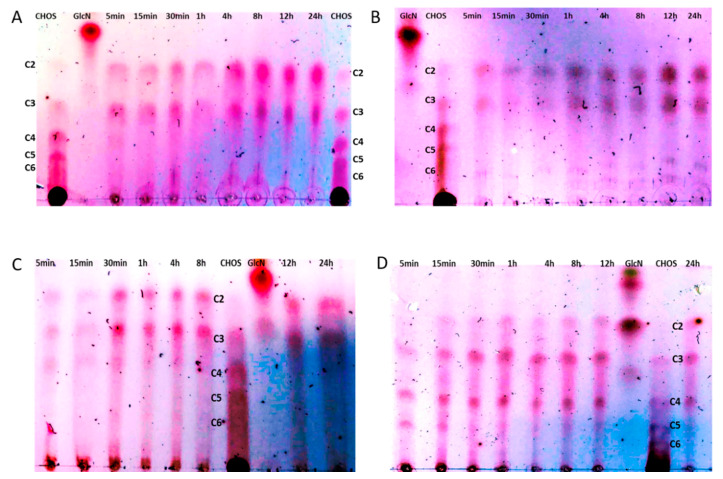
Formation of chito-oligosaccharides (CHOS) from chitosan (0.5% w/v) by chitosanase-displaying *L. plantarum* cells harboring p^alr^Lp0373_CsnA_L at 37 °C (**A**,**B**) and 50 °C (**C**,**D**) using two different substrates: chitosan low molecular weight (**A**,**C**) and chitosan from crab shells medium molecular weight (**B**,**D**) as analysed by thin layer chromatography (TLC). Standards: commercial chito-oligosaccharides (TCI Deutschland GmBH, Eschborn, Germany) which contains chitobiose (**C2**), chitotriose (**C3**), chitotetraose (**C4**), chitopentose (**C5**), chitohexaose (**C6**) and D-glucosamine (GlcN).

Due to their high stability, *L. plantarum* TLG02 cells harbouring the plasmid p^alr^Lp_0373_CsnA_L were selected for enzymatic hydrolysis of chitosan at 37 °C and 50 °C. Chitosan solutions were prepared from two different sources, low molecular weight and chitosan from crab shells medium molecular weight, as described in the materials and methods. The conversion of chitosan to chito-oligosaccharides (CHOS) by CsnA-displaying cells was analyzed by TLC analysis. Analysis of product formation by TLC revealed that surface-displayed chitosanase can degrade low and medium molecular weight chitosan into CHOS (Figure 6A–D). However, high molecular weight chitosan was hardly hydrolysed (data not shown). Chitobiose (C2) and chitotriose (C3) are main products from the conversions of both substrates (Figure 6D). Chitotetraose (C4) was found in the reaction mixtures from the conversions of both substrates after 5 min of the conversions at 50 °C (Figure 6C,D) and it was further degraded with prolonged incubation time in the reaction with low molecular weight substrate (Figure 6C). Interestingly, chitopentaose (C5) was present in the reaction mixture with medium molecular weight substrate at the beginning of the conversion at 50 °C and was then hydrolysed after 15 min (Figure 6D).

## 3. Discussion

In present study, we constructed an expression system for the surface display of a chitosanase from *B. subtilis* in *L. plantarum* based on the pSIP603 expression vector with *alr* gene as the selection marker [27] and the newly constructed system was compared with the original *erm*-based expression system. Two truncated forms S/L with corresponding lengths of 85 and 195 residues from an LP × TG anchor derived from the Lp_1229 sequence were employed to anchor the chitosanase to the cell wall of *L. plantarum*. The S-anchor contains the predicted proline-rich region (PxxP repetitive sequences) located upstream of the C-terminal LPQTNE anchoring motif, whereas the L-anchor extends further and consists of the Mub B2-like domain (mucus binding domain) (Figure 1), which is supposed to generate flexibility of the protein chain and play an important role in the adherence of LAB to the mucus layer covering the epithelial cells of the intestine [29,36]. It was expected that, with the inclusion of the Mub B2-like domain in the linker of L-anchor, CsnA would be more exposed at the surface. Surprisingly, recombinant strains harbouring both the *erm*- and the *alr*-based expression plasmids with the short anchor S resulted in significantly higher chitosanase activities than the long anchor L (*p* < 0.001 with both *alr*- and *erm-*based systems). The activity of surface-displayed enzyme decreases as the length of the anchor is increased. Nevertheless, the attachment of chitosanase on *L. plantarum* cells via the short anchor S was shown to be significantly less stable compared with the long anchor L after several repeated reaction cycles (*p* < 0.05 with *erm-*based system and *p* < 0.001 with *alr-*based system). It could be an explanation for the observations from flow cytometry and immunofluorescence microscopy analyses that the fluorescence signals obtained from the cells harbouring the plasmids with the long anchor L were stronger than those with the short anchor S. Several washing steps required during the preparation for immuno-detection may result in the release of chitosanase from lactobacillal cells carrying the plasmids with the short anchor S. As a result, the long anchor L demonstrates more stable surface localization of chitosanase among these two truncated versions of the LP × TG anchor derived from the Lp_1229 sequence. Furthermore, the food-grade *alr*-based pSIP expression system yields significantly higher production of displayed CsnA than the conventional *erm*-based system (*p* < 0.01 with S anchor and *p* < 0.001 with L anchor).

Interestingly, the attachment of chitosanase on *L. plantarum* cells using these Lp_1229 derived anchors, especially the short anchor S, was more efficient than the cell wall anchor (cwa2) derived from the Lp_2578 protein (*p* < 0.001), of which the highest chitosanase activity was 1360 U per gram dry cell weight (~1.7 mg per g dry cell weight) [22]. The long anchor L has almost the same length as cwa2 (194 residues) and the strain carrying the *erm*-based plasmid, pLp0373_CsnA_L, showed no significant difference in displayed-chitosanase activity, whereas the strain carrying the *alr*-based plasmid, p^alr^Lp0373_CsnA_L, had significantly higher displayed chitosanase activity compared to the cwa2 (*p* < 0.01). However, it should be noted that the plasmid constructed for surface display of CsnA using cwa2 contain N-terminal signal peptide derived from the gene encoding Lp_3050 [22], which is different from the signal peptide Lp0373 used in this study.

## 4. Materials and Methods

### 4.1. Bacterial Strains and Chemicals

The bacterial strains used in this study are listed in Table 1. *Lactobacillus plantarum* WCFS1, isolated from human saliva as described by Kleerebezem et al. [31] was originally obtained from NIZO Food Research (Ede, The Netherlands) and maintained in the culture collection of the Norwegian University of Life Sciences (NMBU), Ås, Norway. *L. plantarum* WCFS1 and TLG02 were grown in deMan, Rogosa and Shape (MRS) broth without/with the addition of 200 μg/mL of D-alanine (Sigma), respectively, at 37 °C without agitation. *Escherichia coli* HST08 (Clontech, Mountain View, CA, USA) and *E. coli* MB2159 used in the transformation experiments involving the subcloning of DNA fragments were cultivated in Luria-Bertani (LB) broth without/with the addition of 200 µg/mL of D-alanine, respectively, at 37 °C with agitation. The agar plates were prepared by adding 1.5% agar to the respective media. When needed, erythromycin was supplemented to media to final concentrations of 5 μg/mL for *L. plantarum* and 200 μg/mL for *E. coli*.

### 4.2. DNA Manipulation

Plasmids were isolated from *E. coli* strains using the Monarch plasmid miniprep kit (New England Biolabs, Frankfurt am Main, Germany), following the instructions of the manufacturers. PCR products and digested fragments were purified using the Monarch DNA Gel extraction kit (New England Biolabs, Frankfurt am Main, Germany) and the DNA amounts were estimated by Nanodrop 2000 (Thermo Fisher Scientific, Waltham, MA, USA). DNA amplifications were performed using Q5^®^ High-Fidelity DNA Polymerase (New England Biolabs, Frankfurt am Main, Germany) and the primers listed in Table 2. Sequences of PCR generated fragments were verified by DNA sequencing performed by a commercial provider (Microsynth, Vienna, Austria). The digestion by restriction enzymes (New England Biolabs) and the ligation of DNA fragments by In-fusion HD Cloning kit (Clontech, Mountain View, CA, USA) were performed following the instructions of the manufacturers. The plasmids with erythromycin resistance gene (*erm)* and the plasmids with alanine racemase gene (*alr*) as the selection markers were transformed into *E. coli* HST08 and *E. coli* MB2159 chemical competent cells, respectively, following the manufacturers’ protocols for obtaining the plasmids in sufficient amounts. The constructed plasmids were transformed into electrocompetent cells of *L. plantarum* WCFS1 or *L. plantarum* TLG02 WCFS1 according to the protocol of Aukrust and Blom [38].

### 4.3. Plasmid Construction

Two truncated forms S and L of the cell wall anchor were derived from Lp_1229, a 1010 amino acid protein encoded by *msa* gene involved in mannose specific adhesion in *L. plantarum* cell wall, which contains LPQTNE motif. The short anchor S comprises 85 C-terminal residues, whereas the long anchor L has the total length of 195 amino residues containing one Mub B2-like domain (mucin binding domain). The anchoring sequences (S, L) used in this study were taken from pLp0373_ManB_S and pLp0373_ManB_L (Table 1), which are derivatives of the pSIP401 vector that has been developed for inducible gene expression in lactobacilli [24]. These pLp0373_ManB_S and pLp0373_ManB_L were used for the construction of the expression plasmids in this study and they all contain a bacterial mannanase gene *manB*, which was fused N-terminally to the signal peptide Lp_0373 [33] and C-terminally to a 30-bp fragment encoding the *myc* tag (GAACAAAAACTCATCTCAGAAGAGGATCTG), followed by the anchoring sequences S and L, respectively.

For construction of the erythromycin-based expression plasmids pLp0373_CsnA_S and pLp_0373_CsnA_L, the *csnA-myc* fragment was generated by three PCR steps using pSIP409_CsnA_native [11] as the template: PCR1 with primers Fwd1_CsnA_SalI and Rev1_CsnA to introduce a N-terminal *Sal*I sites, PCR2 with primers Fwd1_CsnA_SalI and Rev2_CsnA, PCR3 with primers Fwd1_CsnA_SalI and Rev3_CsnA_MluI_S or Rev4_CsnA_MluI_L, which are compatible with short anchor S or long anchor L encoding fragments, respectively, to introduce a C-terminal *Mlu*I sites. The resulting PCR fragments were ligated into the *Sal*I/*Mlu*I digested plasmids: pLp0373_ManB_S and pLp0373_ManB_L using In-Fusion HD Cloning kit (Clontech, Mountain View, CA, USA) resulting in two plasmids pLp0373_CsnA_S and pLp_0373_CsnA_L, respectively (Figure 1A,B).

For the construction of the food-grade expression plasmids, the expression cassettes, P*_sppA_*-SPLp0373-*csnA*-*myc*-S/L (~1.3–1.6 kb, Figure 1A) containing the promoter P*_sppA_* (of pSIP401), the signal peptide Lp_0373, *csnA* gene fused to the *myc* tag followed by one of the anchoring sequences S/L were amplified using the primer pair Fwd2_CsnA_BglII and Rev5_CsnA_S/L_EcoRI and the newly constructed plasmids pLp0373_CsnA_S and pLp0373_CsnA_L as templates. The resulting PCR fragments were then ligated into ~5.8 kb *Bgl*II-*Eco*RI digested fragment of pSIP603 vector, in which the erythromycin resistance gene (*erm)* was replaced by the alanine racemase gene (*alr*) as the selection marker [27], using In-Fusion HD Cloning kit (Clontech, Mountain View, CA, USA) yielding two food-grade expression plasmids named p^alr^Lp0373_CsnA_S and p^alr^Lp_0373_CsnA_L (Figure 1C).

### 4.4. Gene Expression in L. plantarum

The expression plasmids pLp0373_CsnA_S and pLp0373_CsnA_L were constructed in *E. coli* HST08 before electroporation into *L. plantarum* WCFS1 competent cells and transformants were selected on MRS agar plates containing 5 µg/mL erythromycin. To generate the food-grade expression strains, the food-grade expression plasmids p^alr^Lp0373_CsnA_S and p^alr^Lp0373_CsnA_L were constructed in *E. coli* MB2159 before electroporation into electro-competent *L. plantarum* TLG02, a D-alanine auxotroph expression host and the selection of transformants was performed on MRS agar plates.

Gene expression was carried out by diluting the overnight cultures of *L. plantarum* strains harbouring the plasmids in 100 mL of fresh pre-warm MRS broth (for *erm-*based systems, 5 µg/mL of erythromycin was added) to an OD_600_ of ~0.1, and incubated at 37 °C without agitation. The cells were induced at an OD_600_ of 0.3 by adding the peptide pheromone IP-673 [39] to final concentration of 25 ng/mL. Cells were harvested at OD_600_ of ~1.0–1.2 by centrifugation (4000× *g*, 4 °C, 10 min), washed twice with phosphate buffered saline (PBS) containing 137 mM NaCl, 2.7 mM KCl, 2 mM KH_2_PO_4_, and 10 mM Na_2_HPO_4_ (pH 7.4), and then re-suspended in PBS buffer.

### 4.5. Enzymatic Activity Measurement

Chitosanase activity was determined as described previously [10,22] with some modifications. Chitosanase-displaying cells were collected from the cultures by centrifugation at 4000× *g* for 10 min at 4 °C. Cell pellets obtained from cultures were washed twice with PBS and re-suspended in 200 µL of PBS. Chitosan (PT Biotech Surindo, Jawa Barat, Indonesia) was completely dissolved in 1% (w/v) of acetic acid at 80 °C for 30 min before adjusting the pH of the solution to 5.5 with 1 M NaOH. The reaction was conducted with 100 µL of an enzyme-displaying cells suspension in PBS buffer and 400 µL of a 0.5% (w/v) chitosan solution at 37 °C for 5 min with mixing at 800 rpm. The cells and the supernatant were separated by centrifugation (4000× *g*, 4 °C, 2 min). The amount of reducing sugar released in the supernatant of the enzymatic reaction was determined by the dinitroasalicylic acid (DNS) assay. DNS assay was carried out with 100 µL of the reaction supernatant mixed with 100 µL of DNS solution at 99 °C for 10 min, followed by cooling on ice for 5 min. The mixture was then diluted with 800 µL of de-ionised water before measuring the absorbance at 540 nm using 1–5 µmol/mL of D-glucosamine as standards. One unit of chitosanase activity was defined as the amount of enzyme releasing 1 µmol of reducing sugars (or reducing end equivalents) per minute under the given conditions.

### 4.6. Western Blotting

The cells obtained from 50 mL of cultures were disrupted with glass-beads (170 µm; Sigma Aldrich, Darmstadt, Germany) using the Precelly 24 glass bead mill (PEQLAB Biotechnology GmbH, Erlangen, Germany) and cell-free extracts (crude extracts) were obtained after 5 min of centrifugation at 10,000× *g* and 4 °C. Protein concentrations in the cell-free extracts were measured by Bradford assay [40] and separated on SDS-PAGE gels before being transferred to a nitrocellulose membrane using the Trans-Blot Turbo Transfer system (Bio-Rad Laboratories, Hercules, CA, USA) following the instructions of the manufacturer. After nonspecific protein interactions were blocked by incubating the membrane with 50 mL of 1% BSA dissolved in Tris-buffered saline-Tween 20 (TBS-T) for one hour on the shaker at room temperature, the membrane was immediately incubated with 1 µL of monoclonal murine anti-Myc antibody (Invitrogen, Carlsbad, CA, USA; diluted 1:5000) in TBS-T buffer containing 0.5% of BSA at 4 °C overnight. After three times washing with 15 mL of TBS-T buffer, the membrane was incubated with 2.5 µL of a secondary antibody, which was polyclonal rabbit anti-mouse antibody conjugated with horseradish peroxidase (HRP) (Dako, Glostrup, Denmark), diluted 1:2000 in TBS-T buffer containing 0.5% BSA for 1 h in the dark at room temperature. Before visualization, the membrane was rinsed again three times with 15 mL of TBS-T, following by incubation with a Clarity Western ECL Blotting Substrate (Bio-Rad Laboratories, Hercules, CA, USA). The protein bands were visualized by the ChemiDoc™ XRS+ imaging system (Bio-Rad Laboratories, Hercules, CA, USA).

### 4.7. Flow Cytometry Analysis

Cell staining for flow cytometry was carried out as previously described [22] with some modifications. One mL of cell culture (OD_600_ of ~0.5) was obtained 2 h after induction, and the cells were incubated in 50 µL PBS containing 2% of BSA (PBS-B) with 0.1 µL monoclonal anti-Myc antibody (Invitrogen, Carlsbad, CA, USA; diluted 1:250 in PBS-B) at room temperature for 40 min. Subsequently, the cell suspension was centrifuged at 5000× *g* for 3 min at 4 °C, washed three times with 500 µL PBS-B, and then incubated with 0.2 µL anti-mouse IgG antibody (Alexa Fluor 488 conjugated; Cell Signalling Technology, Frankfurt am Main, Germany) diluted 1:500 in 50 μL PBS-B for 40 min at room temperature, in the dark. After collecting the cells by centrifugation (4000× *g*, 3 min at 4 °C) and washing three times with 500 µL PBS-B, the stained cells were resuspended in 100 µL of PBS and analysed using a Gallios Flow cytometer (Beckman Coulter, Brea, CA, USA), following the manufacturer’s instructions. The data were analysed by Kaluza Analysis software (Beckman Coulter, Brea, CA, USA).

### 4.8. Indirect Immunofluorescence Microscopy Analysis

Cell fixation and staining for indirect immunofluorescence microscopy were carried out as previously described [22,41,42,43] with some modifications. One mL of cell culture (OD_600_ of ~0.5) was harvested 2 h after induction by centrifugation at 4000× *g* for 10 min at 4 °C, and re-suspended in 100 µL PBS (pH 7.4). The cells were fixed with ethanol to final concentration of 70% (w/w) for 1 h at −20 °C [41,42]. Fixed cells were washed twice with PBS and was resuspended in 100 μL of PBS. An amount of 30 μL of fixed cells was transferred onto a microscope slide and the cells were absorbed on the slide for 2 h at room temperature until the trace was dried. Fixed cells were then incubated with 100 µL of PBS containing 10% of BSA for 1 h to block nonspecific protein interactions. Subsequently, the cells were stained with anti-Myc antibody (Invitrogen, Carlsbad, CA, USA) and anti-mouse IgG antibody (Alexa Fluor 488 conjugated; Cell Signalling Technology, Frankfurt am Main, Germany) as described above. The stained cells were washed three times with 100 μL of PBS (5 min for each washing step) and the slide was mounted with 5 μL of mounting medium containing 50% glycerol in PBS [43]. The stained cells were observed under a Leica DMI6000B ‘Live cell’ wide-field fluorescence microscope (Leica Microsystems; Wetzlar, Germany) using the 488-nm argon laser line. The fluorescence detection window was set between 505 nm and 550 nm and the images were acquired with a PL APO 63×/1.40 oil immersion objective.

### 4.9. Catalytic Stability and Thermal Stability of Chitosanase Displaying Cells

Chitosanase displaying cells were collected from cultures by centrifugation at 4000× *g* for 10 min at 4 °C. Cell pellets were washed twice with PBS and re-suspended in 100 µL of PBS and chitosanase activities were measured at 37 °C as described above. This procedure was repeated for several cycles of activity measurements with intermediate two washing steps to determine the number of use cycles of surface displayed chitosanase.

In order to determine the thermal stability of chitosanase displaying cells at various temperatures (−20, 37, and 50 °C), *L. plantarum* TLG02 cells harbouring p^alr^Lp_0373_CsnA_L were collected from 20 mL culture by centrifugation at 4000× *g* for 10 min at 4 °C and re-suspended in 1 mL of PBS prior to incubation at these temperatures. At certain time intervals, the enzymatic activity of chitosanase-displaying cells was measured using chitosan as the substrate under standard assay conditions. The half-life value (τ _½_) of activity was determined when residual activity reaches 50%.

### 4.10. Chitosan Conversion

Conversion of 0.5% (w/v) chitosan low molecular weight and chitosan from crab shells medium molecular weight, which were prepared in 1% (w/v) of acetic acid at 80 °C for 30 min before adjusting the pH of the solution to 5.5 with 1 M NaOH, catalysed by surface displayed chitosanase was carried out on a 2-mL scale with 5 U of chitosanase per mL of reaction mixture at 37 °C and 50 °C for 24 h. Surface-displayed chitosanase was obtained from the expression strain *L. plantarum* TLG02 harbouring the plasmid p^alr^Lp_0373_CsnA_L. Agitation was applied at 150 rpm and the samples were taken at regular intervals. The reactions were stopped by heating the samples at 99 °C for 5 min prior to further analyses.

### 4.11. Thin Layer Chromatography (TLC) Analysis

TLC was performed by high performance TLC (HPTLC) silicagel plate (Kiselgel 60 F245, Merck, Kenilworth, NJ, USA) as previously described [10,44] with some modifications. Approximately 0.5 µL of samples were applied to the plate and eluted twice in ascending mode with an iso-propanol/25% ammonia/water mixture (7:1:2). Thymol reagent was used for visualization. A commercial chito-oligosaccharides (TCI Deutschland GmBH, Eschborn, Germany) with a mixture of C2–C6, which contains chitobiose, chitotriose, chitotetraose, chitopentose, chitohexaose, and D-glucosamine (GlcN) (Sigma Aldrich, Darmstadt, Germany) were used as standards.

### 4.12. Statistical Analysis

All measurements were conducted in triplicates, and the standard deviation was always less than 5%. Student’s *t* test was used for the comparison of data.

## 5. Conclusions

We have demonstrated the successful anchoring of a bacterial chitosanase onto the cell surface of *L. plantarum* using a food-grade lactobacillal expression system and two truncated forms of the LP × TG cell wall anchor derived from the Lp_1229 sequence. CsnA displayed on *L. plantarum* cells is catalytically active and can convert chitosan into chito-oligosaccharides, of which chitobiose and chitotriose are the main products. The successful development of the food-grade *alr-*based expression system, which overcomes certain drawbacks of the original *erm*-based system, for the surface display of an active chitosanase in *L. plantarum* will certainly result in a stable, food-grade, whole-cell biocatalyst that could be of interest for the production of oligosaccharides of prebiotic potential.

## Figures and Tables

**Table 1:** Strains and plasmids used in this study.

Strain or Plasmid	Relevant Characteristic (s)	Reference Source
Strains		
*L. plantarum*		
WCFS1	wild type, host strain	[31]
TLG02	Δ*alr*, D-alanine auxotroph, food-grade expression host	[27]
*E. coli*		
HST08	cloning host	Clontech
MB2159	D-alanine auxotroph, cloning host	[37]
Plasmids		
pLp0373_ManB_S	Erm^r^; pSIP401 derivate encoding the Lp_0373 signal peptide translationally fused to *manB-myc*, followed by the short cell wall anchor (S) from Lp_1229	(unpublished)
pLp0373_ManB_L	Erm^r^; pSIP401 derivate encoding the Lp_0373 signal peptide translationally fused to *manB-myc*, followed by the long cell wall anchor (L) from Lp_1229	(unpublished)
pSIP409-CsnA-native	Erm^r^; *spp*- based expression vector pSIP409 for expression of *csnA* with native signal peptide	[11]
pEV	Erm^r^; pSIP401 derivative, empty vector, no signal sequence, no *csnA* (negative control)	[13]
pSIP603-GusA	Erm^r^; pSIP401 derivative, *gusA* controlled by P*_sppA_*, *alr* replaced *erm*	[27]
pLp0373_CsnA_S	Erm^r^; pLp0373_ManB_S derivative with *csnA-myc* instead of *manB-myc*	This study
pLp0373_CsnA_L	Erm^r^; pLp0373_ManB_L derivative with *csnA-myc* instead of *manB-myc*	This study
p^alr^Lp0373_CsnA_S	pSIP603 derivative with SPLp0373-*csnA*-*myc*-S instead of *gusA*	This study
p^alr^Lp0373_CsnA_L	pSIP603 derivative with SPLp0373-*csnA*-*myc*-L instead of *gusA*	This study

**Table 2:** Primers used in this study.

Primer	Sequence ^a^ 5′ → 3′	Restriction Site Underlined
Fwd1_CsnA_SalI	TGCTTCATCAGTCGAC*GCGGGACTGAATAAAGATC*	*Sal*I
Fwd2_CsnA-BglII	ATTACAGCTCCAGATCT*ACCGGTGGGCC*	*Bgl*II
Rev1_CsnA	TGAGATGAGTTTTTGTTC*GTCGACAGATCCTTTGATTAC*	
Rev2_CsnA	CAGATCCTCTTC*TGAGATGAGTTTTTGTTCGTCGACAGA*	
Rev3_ CsnA_MluI_S	CTGGTTTAACACGCGT*CAGATCCTCTTCTGAGATG*	*Mlu*I
Rev4_CsnA_MluI_L	GAGCATTCTTGGTACGCGT*CAGATCCTCTTC*	*Mlu*I
Rev5_CsnA_S/L_EcoRI	GGGGTACCGAATTC*AAGCTTCTACTCTTTGTGCTGTC*	*EcoR*I

^a^ The nucleotides in italics are the positions that anneal to the DNA of the target gene.
